# Oronasal fistula in cleft palate surgery

**DOI:** 10.4103/0970-0358.57203

**Published:** 2009-10

**Authors:** Partha Sadhu

**Affiliations:** Global Hospital & Research Centre, Mount Abu, India

**Keywords:** Cleft palate, Oronasal fistula, Palatal fistula

## Abstract

Oronasal fistula (ONF) is the commonest complication associated with cleft palate surgery. The main symptoms associated with ONF are nasal regurgitation of food matter and hypernasality of voice. Repair of cleft palate under tension is considered to be the main reason of ONF though vascular accidents and infection can also be the cause. Most of the ONFs are situated in the hard palate or at the junction of hard and soft palate. Repair of ONF depends on its site, size and mode of presentation. A whole spectrum of surgical procedures starting from small local flaps to microvascular tissue transfers have been employed for closure of ONF. Recurrence rate of ONF is 25% on an average after the first attempt of repair.

## INTRODUCTION

Oronasal fistula (ONF) is probably the commonest complication associated with cleft palate surgery. The rate of ONF varies from 4-35%[1] or even more in case of primary palatoplasty. The two main symptoms associated with ONF are nasal regurgitation and speech problems, mainly hypernasality. The site and size of the fistula are variable and so are the causes. ONF develops primarily because of repair under tension and in some cases, especially in adults, as a result of postoperative infection.

As already mentioned, the incidence is highly variable. Musgrave and Bremmer[2] presented healing problems in about 10% of their cases where approximately 7% developed fistulas. They found, the incidence to be more in bilateral (12.5%) than in unilateral cases (7.7%). Kilner[3] reported only a rare failure of union and he almost always thought it was due to infection, whereas Holdsworth[4] felt that wound infection is surprisingly rare. In recent days, Phua and de Chalain[5] in a study of 211 patients collectively operated by five different surgeons found an overall fistula rate of 12.8% over a mean follow-up period of four years 10 months. Fistula rates were higher for the more severe degree of clefting but were not affected by gender or type of surgical repair.

## AETIOLOGY OF ONF

**Figure 1:**
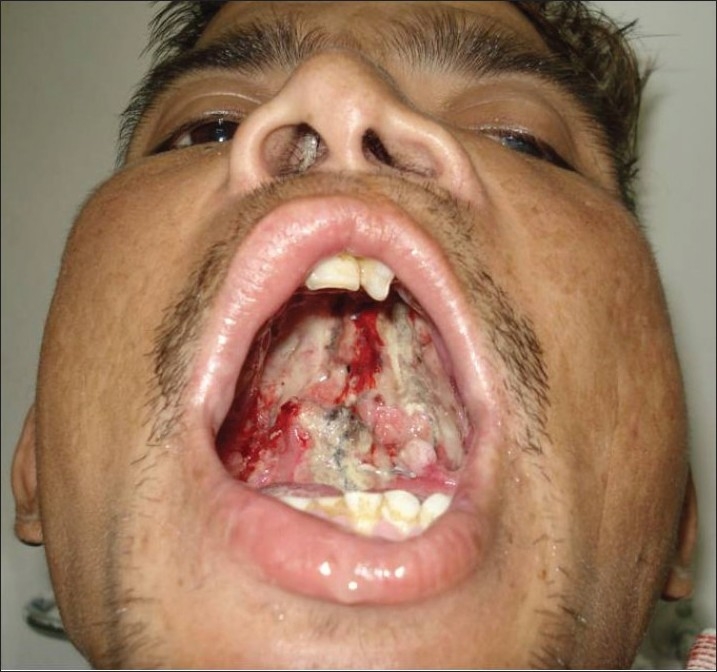
Bilateral damage to greater palatine arteries. Flaps necrosed

The primary cause of development of ONF is repair under tension. However, there are some palatal clefts which are quite wide and the available tissue to repair the palate seems inadequate. In these cases, the chance of development of ONF is higher though in experienced hands they can be prevented. The other reason is postoperative infection which is hardly seen in small children. The protocol of swab-culture from pharyngeal wall has not been adopted by the author. Vascular accidents during palatoplasty can cause flap loss and is relatively an uncommon reason for development of ONF [Figure 1]. Besides these, inadvertent use of diathermy, particularly near the greater palatine pedicle can compromise the blood supply of the mucoperiosteal flap and can result in an ONF.

## CLASSIFICATION OF ONF

Based on their size, fistulas may be classified as small (< 2mm), medium (3-5mm) or large (>5mm).[6]

According to the location, fistulas are described as anterior fistula, midpalatal fistula, fistula at the junction of the soft palate and hard palate and soft palate fistula. In a study of 64 patients, Diah, Lo, Yun *et al*.[7] reported the hard-soft plalte junction as the commonest site (53.1%). Local flaps and two-flap palatoplasty were the most common techniques used to repair these ONFs. They also reported that 25% of these cases were reoperated for recurrence of the fistula. Similar site and rate of occurrence was reported by Amartunga.[8]

## SURGICAL TREATMENT OF ONF

Timing of surgery: Surgical closure of ONF should be attempted at least six months after the previous surgery.

**Figure 2:**
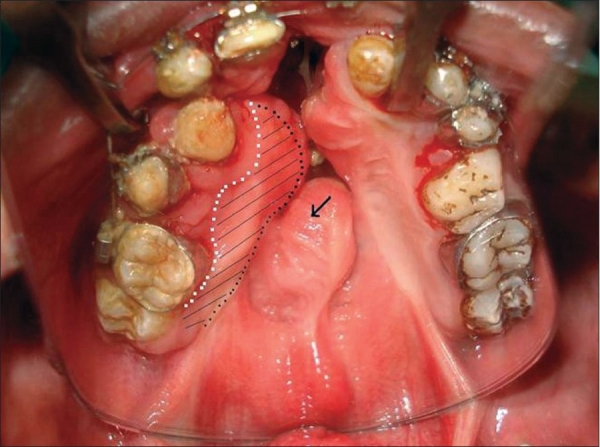
Previous incision mark and anterior rugosity

Assessment: Other than the size and site of the ONF the important factor in assessment is the amount of scar tissue present around the fistula. A close inspection will reveal the previous incision mark if used for lateral release. Lateral to this mark is all epithelialised scar tissue which if elevated as flap has unpredictable vascular supply [Figure 2]. If the previous surgery was done a long time ago, this tissue may behave like a normal mucoperiosteal flap. It is wise to elevate the mucoperiosteal flap from the crevicular margin to ensure greater width and length of the flap. The rugosity [Figure 2] that is present in the anteriormost aspect of the mucoperiosteal flap should be inspected. Presence of this landmark usually excludes vascular accidents in previous surgery. If this is found to be situated almost in the mid-palatal region, it indicates the oral flaps have gone into significant contracture. In case where the ONF is quite big and a vascular accident is suspected, a handheld Doppler probe can be used before making the incision to assess the greater palatine pedicle. If no signal is registered by the probe, it is better not to elevate the palatal mucoperiosteum on that side.

### Surgical principle

Preferably the fistula should be closed in two layers. Both the layers should have well-vascularised tissue and the suturing should be free of tension. There are also reports in literature wherein closure of ONF was effected in three layers.[9] As an intermediate layer, cartilage, bone and acellular dermal matrix have been used.

### Surgical techniques

**Figure 3:**
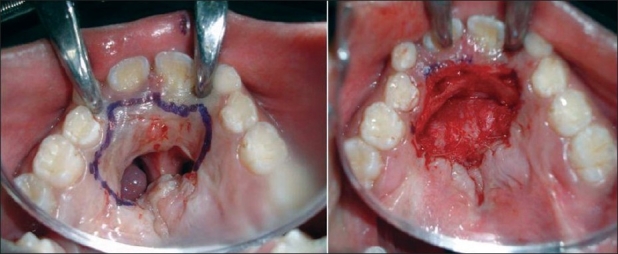
Anterior fistula with intact alveolus. Single-layer closure by turnover flap (not recommended

Closure by small local flaps: Small ONF can be closed by local flaps only. A turnover flap is developed from the mucoperiosteal layer on the non-cleft side (in case of unilateral cleft) to make the nasal lining. Another rotation flap is developed from the other mucoperiosteal layer to create the oral layer. The rotation flap required is usually bigger than expected. Single-layer closure can be achieved in selected cases. In that case a bigger turnover flap is harvested and is tucked under the opposite mucoperiosteal layer in a double-breasting technique [Figure 3]. The recurrence rate in single-layer closure is higher.

### Redo palate

**Figure 4:**
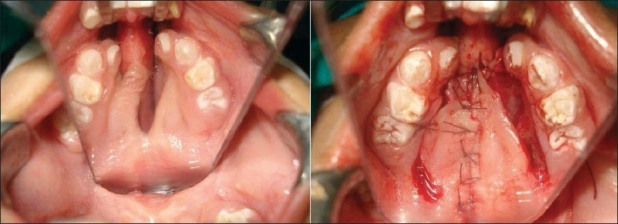
Anterior fistula. Redo of the hard palate

This is an option for mid-palatal ONF surrounded by adequate palatal tissue and is associated with velopharyngeal incompetence. A complete redo palatoplasty addresses both the problems [Figure 4].

### Use of a buccal mucosal flap

**Figure 5:**
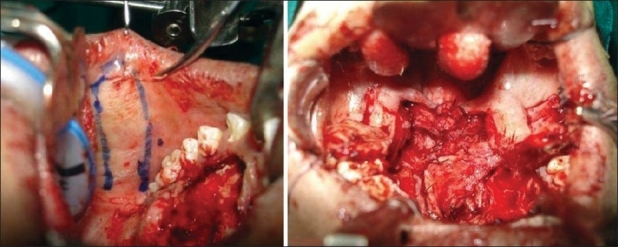
Buccal mucosal flap marking and inset

Originally described as a cheek flap by Mukherji[10] who used it in primary palatoplasty for short palates, this is particularly helpful when the fistula is situated near the hard palate - soft palate junction and the original cleft is also wide. Buccal mucosal flap is a posteriorly based, random pattern flap with its base situated near the retromolar trigone [Figure 5]. The distal end of the flap can be harvested to a point little short of the oral commissure and the only structure to be taken care of during flap harvest is the papilla of the parotid duct. The average width of the flap is 15 mm in children. However, a wider flap can be harvested in young adults. The flap, if necessary, can be harvested from both sides and can be used both for oral as well as the nasal layer. Part of the buccinator muscle can be incorporated in the flap too to make it more reliable and robust. In that case, the flap is termed as buccinator myomucosal flap.

### Mucosal/myomucosal flap from the under-surface of the lip [Figure 6]:

**Figure 6:**
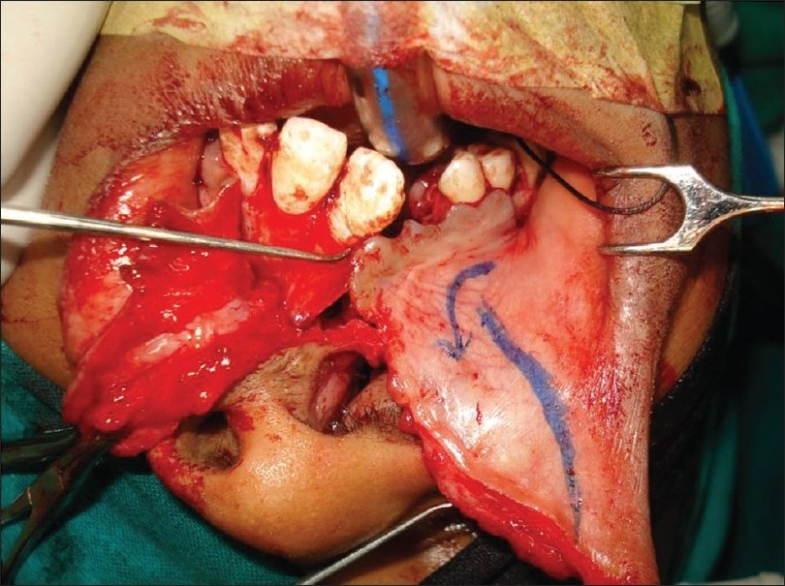
Myomucosal flap from the under-surface of the lip

**Figure 7:**
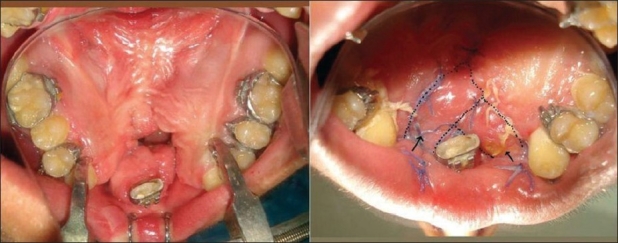
Bilateral anterior fistulas. Use of bilateral flaps from the undersurface of the lip around the premaxilla

This is particularly helpful for anterior fistulas where there is deficiency in oral mucoperiosteal layer. The flap can be transported into the palate through the alveolar cleft and can reach 3-4 cm into the palate. Similar flaps can be taken bilaterally in case of bilateral cleft lip and palate to close anterior fistulas on either side of the premaxilla [Figure 7].

### Tongue flap

This is a two-stage procedure. Use of lingual tissue in palatal fistula repair has been reported more than 50 years ago by Santos and Altamirano.[11] Jackson[12] has shown that the flap is safe and well tolerated by children when executed properly. The flap is indicated for bigger ONF where there is significant tissue deficit in the oral mucoperiosteal layer. The flap can be anteriorly [Figure 8a,b] or posteriorly based depending on the site of the fistula. Composition-wise, this is a myomucosal flap and the average thickness should not be less than 6 mm to ensure its vascularity. A good nasal layer repair is a prerequisite for success. Detachment is done on the 10^th^ to 14^th^ postoperative day. Children tolerate the flap quite well and usually there is no need to put a naso-gastric tube for feeding. The donor area is almost always closed primarily and there is no residual defect of the tongue or any speech problem.

**Figure 8a:**
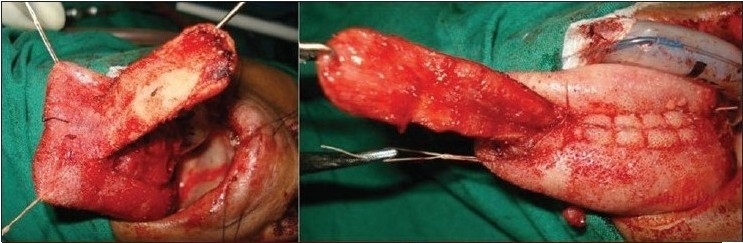
Anteriorly based tongue flap and closure of the donor site

**Figure 8b:**
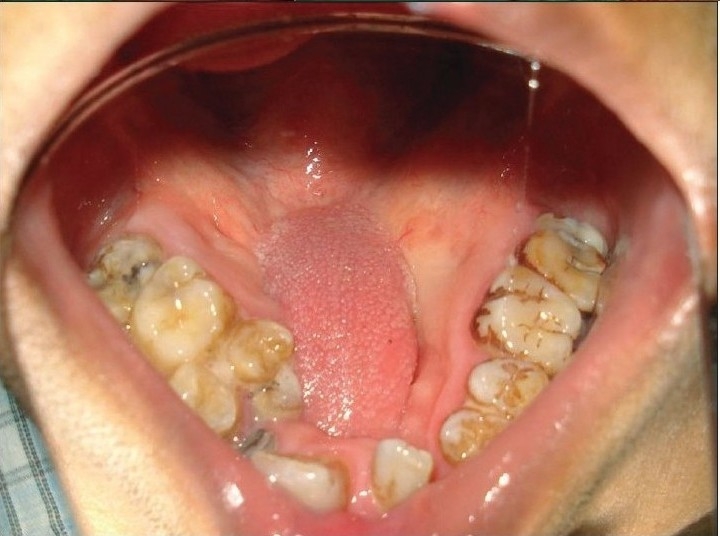
Repaired fistula by tongue flap

The two main complications are haemorrhage and spontaneous detachment from the palate. A pre-fabricated ‘flap retainer’ can be used to ensure the success of the flap.[13]

### Facial artery myomucosal flap (FAMM)

The flap was described by Pribaz *et al*.[14] in 1992. This flap is particularly useful for bigger ONF in the anterior palate which is extending to the mid-palatal region and is associated with an alveolar cleft. The facial artery of the cleft side is traced with a handheld Doppler at the beginning of the surgery. The base of the flap is near the alar base. The width can be up to 2.5 to 3 cm and is myomucosal in composition. The facial artery is divided as it crosses the lower border of the mandible and is incorporated in the flap as its central axis. Preferably, the nasal lining should be created with local tissue on top of which the flap is inset. The alveolar cleft is the gateway for this flap to enter from the cheek to the palate.

### Free tissue transfer

Free radial forearm flap,[15] and free scapular flap have been documented to be used to close big ONF where local tissue is not available. The composition of the flap can be adipocutaneous, fasciocutaneous or adipofascial depending on the choice of the surgeon.

Besides these, the use of the turbinate flap has been reported by Penna *et al*.[16] Temporoparietal galeal flap has been also successfully used for closure of ONF.[17] Losee *et al*.[18] published the use of acellular dermal matrix in 39.4% cases of primary palatoplasty to prevent ONF.

### Nonsurgical closure

**Figure 9:**
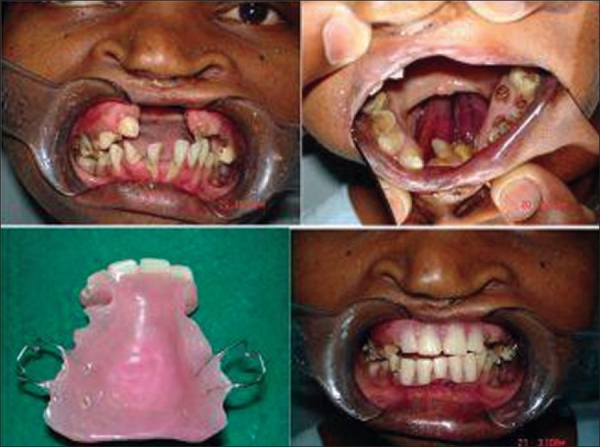
Prosthetic management of ONF

The ONF can be closed by a plate designed by an orthodontist colleague. This is indicated where the patient has gone through several unsuccessful surgical attempts and is no longer willing to undergo another procedure. This plate can have an alveolar component which bridges the alveolar cleft and can have artificial teeth incorporated in it [Figure 9].

### Author's experience

The author performs primary palatoplasty by three different techniques. They are a) Bardach's two-flap palatoplasty, b) Furlow's technique and c) two-stage palatoplasty where the hard palate is closed by single-layer vomer flap during lip repair and soft palate is closed at a later date. Considering all three procedures, we have a very low fistula rate of around 1%. We never perform a preoperative throat swab culture and the small percentage of fistulas that we have, we found the culture negative in the postoperative period. We firmly believe that ONF is almost always related to the surgical technique.

We describe the fistulas in the following way:

- Isolated small to medium-sized ONF
- ONF with velopharyngeal incompetence (VPI)
- ONF with a repaired lip-nose that needs revision surgery
- ONF in the alveolar region presenting at mixed dentition period
- Huge ONF

**Figure 10:**
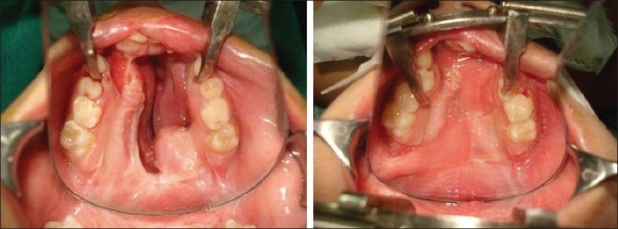
Huge ONF repaired with combination of intraoral flaps

Simple ONF is repaired most of the time by local flaps or by redo of the hard palate. Single-layer repair is not advocated because of the high rate of recurrence. For ONF with VPI a complete redo palatoplasty is done which addresses both the problems in the same sitting. Sometimes, a buccal mucosal flap is incorporated in the nasal layer of the soft palate which helps in lengthening and creates a tension-free repair. In cases where the lip needs a revision surgery, it is always advisable to open the lip and do a revision along with the ONF repair. The access to the fistula becomes more direct and the nasal floor repair becomes easier and better. For patients with an anterior fistula, who present during mixed dentition period, we prefer to prepare the child orthodontically and to perform an alveolar bone graft in the same sitting. It is in huge ONF usually caused by vascular accidents that a tongue flap in combination with other flaps needs to be used. However, every case of ONF has to be individualised and accordingly the treatment plan has to be made. We prefer to wait for 10-12 months after the previous surgery, unless there is strong recommendation from the speech pathologist for early closure. The author has very limited experience with tongue flaps and has never performed free tissue transfer for palatal fistula closure. We have successfully closed palatal defects by temporalis muscle but not in a cleft patient. In one case of recurrent fistula where half the hard palate was destroyed, we closed it with a combination of intraoral flaps. After repairing the nasal layer by mobilization of available local tissue, a FAMM flap from one cheek and a buccal mucosal flap from the other cheek were used to close this big ONF [Figure 10,b].

## RESULTS

With a single-layer closure, the recurrence rate of fistulas was about 40%; hence a single-layer closure should be avoided. The rate of recurrence for hard palate ONF after complete redo of hard palate is less than 5%. Recurrence of ONF at the junction of hard and soft palate is nil. This could be attributed to frequent use of buccal mucosal flap to repair these fistulas.

## CONCLUSION

Oronasal fistula is the commonest complication of cleft palate surgery. The incidence is highly variable though almost always the primary cause remains the same i.e. closure under tension. Symptomatic ONF is associated with nasal regurgitation and hypernasality of speech. Principle of repair of ONF is apposition of well-vascularised tissue without tension. Different techniques, starting from local flaps to free tissue transfer have been employed to repair ONF depending on its site, size and tissue available. In general the recurrence rate of ONF is around 25%. With better technique and skill, the incidence and recurrence rate of ONF can both be minimized.

## References

[1] Cohen SR, Kalinowski J, LaRossa D, Randall P (1991). Cleft palate fistulas: A multivariate statistical analysis of prevalence, etiology, and surgical management. Plast Reconstr Surg.

[2] Musgrave RH, Bremner JC (1960). Complications of cleft palate surgery. Plast Reconstr Surg.

[3] Kilner TP (1937). Cleft lip and palate repair technique. St Thomas's Hosp Report.

[4] Holdsworth WG (1957). Cleft lip and palate.

[5] Phua YS, de Chalain T (2008). Incidence of oronasal fistulae and velopharyngeal insufficiency after palate repair: An audit of 211 children born between 1990 and 2004. Cleft palate Craniofac J.

[6] Muzaffar AR, Byrd HS, Rohrich RJ, Johns DF, LeBlanc D, Beran SJ (2001). Incidence of cleft palate fistula: An institutional experience with two stage palate repair. Plast Reconstr Surg.

[7] Diah E, Lo LJ, Yun C, Wang R, Wahyuni LK, Chen YR (2007). Cleft oronasal fistula: A review of treatment results and a surgical management algorithm proposal. Chung Gang Med J.

[8] Amaratunga NA (1988). Occurrence of oronasal fistula in operated cleft palate patients. J Oral Maxillofacial Surg.

[9] Murrell GL, Requena R, Karakla DW (2001). Occurrence of oronasal fistulas in operated cleft palate patients. Plast Reconstr Surg.

[10] Mukherji MM (1969). Cheek flap for short palates. Cleft Palate Craniofac J.

[11] Guerrero-Santos J, Altamirano JT (1966). The use of lingual flaps in repair of fistulae of the hard palate. Plast Reconstr Surg.

[12] Jackson IT (1972). Closure of secondary palatal fistulae with intraoral tissue and bone grafting. Brit J Plast Surg.

[13] Agarwal K, Panda K (2007). Management of a detached tongue flap. Plast Reconstr Surg.

[14] Pribaz J, Stephens W, Crespo L, Gifford G (1992). A new intraoral flap: Facial artery musculomucosal (FAMM) flap. Plast Reconstr Surg.

[15] Chen HC, Ganos DL, Coessens BC, Kyutoku S, Noordhoff MS (1992). Free radial forearm flap for closure of difficult oronasal fistulas in cleft palate patients. Plast Reconstr Surg.

[16] Penna V, Bannasch H, Stark GB (2007). The turbinate flap for oronasal fistula closure. Ann Plast Surg.

[17] Fallah DM, Baur DA, Ferguson HW, Helman JI (2003). Clinical application of the temporoparietal-galeal flap in closure of a chronic oronasal fistula: review of the anatomy, surgical technique, and report of a case. J Oral Maxillofac Surg.

[18] Losee JE, Smith DM, Afifi AM, Jiang S, Ford M, Vecchione L (2008). A successful algorithm for limiting postoperative fistulae following palatal procedures in the patient with orofacial clefting. Plast Reconstr Surg.

